# Long‐term observation of pulmonary toxicity of toner with external additives following a single intratracheal instillation in rats

**DOI:** 10.1002/1348-9585.12146

**Published:** 2020-07-25

**Authors:** Taisuke Tomonaga, Hiroto Izumi, Yukiko Yoshiura, Takashi Marui, Ke‐Yong Wang, Chinatsu Nishida, Kazuhiro Yatera, Yasuo Morimoto

**Affiliations:** ^1^ Department of Occupational Pneumology Institute of Industrial Ecological Sciences University of Occupational and Environmental Health Kitakyushu Fukuoka Japan; ^2^ Shared‐Use Research Center School of Medicine University of Occupational and Environmental Health Kitakyushu Fukuoka Japan; ^3^ Department of Respiratory Medicine University of Occupational and Environmental Health Kitakyushu Fukuoka Japan

**Keywords:** DNA damage, external additives, intratracheal instillation, rat, toner, tumorigenesis

## Abstract

**Objectives:**

Along with technological innovations for improving the efficiency of printing, nanoparticles have been added to the surface of toners, and there is concern about the harmful effects of those components. We investigated, through a long‐term observation following intratracheal instillation using rats, whether exposure to a toner with external additives can cause tumorigenesis.

**Methods:**

Female Wistar rats were intratracheally instilled with dispersed toner at low (1 mg/rat) and high (2 mg/rat) doses, and the rats were sacrificed at 24 months after exposure, after which we examined pulmonary inflammation, histopathological changes, and DNA damage in the lung. Rats that had deceased before 24 months were dissected at that time as well, to compare tumor development.

**Results:**

Although alveolar macrophages with pigment deposition in the alveoli were observed in the 1 and 2 mg exposure groups, no significant lung inflammation/fibrosis or tumor was observed. Since immunostaining with 8‐OHdG or γ‐H2AX did not show a remarkable positive reaction, it is thought that toner did not cause severe DNA damage to lung tissue.

**Conclusion:**

These results suggest that toner with external additives may have low toxicity in the lung.

## 1 BACKGROUND

Toner is used in printers in offices and homes everywhere, and it is indispensable in daily life, although there are various reports on the harmful effects of, mainly, carbon black, which is a main component of toner.^1^, ^2^, ^3^, ^4^ Along with technological innovations for improving the efficiency of printing, nanoparticles like titanium dioxide and silica have recently been added to the surface of toners. There are some reports that these new toners give off volatile organic compounds (VOC) and nanoparticles in the air as external additives.^5^, ^6^ Recent years, biological adverse effects by nanoparticles are concerned.^7^, ^8^, ^9^, ^10^ It has been reported that nanometer‐sized particles generally cause greater pulmonary inflammation than micrometer‐sized particles,^9^, ^10^ and it is therefore thought that adding nanoparticles to toners could cause pulmonary toxicity.

Lung tumor is an irreversible pathological change in lung disorders induced by inhaled chemicals. Generally, in lung disorders caused by inhalant dust, infiltration of neutrophils, and alveolar macrophages induce pulmonary inflammation, and persistent or progressed inflammation is likely to lead to irreversible fibrosis and eventually cause tumors.^11^ It is known that materials with high toxicity, such as silica and asbestos, lead to persistent inflammation and cause fibrosis and tumors,^3^, ^12^ whereas it is also reported that materials with low toxicity, such as titanium dioxide and fullerene, do not proceed irreversibly because of transient changes, even if inflammation occurs.^13^, ^14^ Thus, persistent inflammation might form the basis of tumor development through fibrosis. It is thought that carcinogenicity involves a series of events that include genetic mutations, changes in oncogenes and tumor suppressor genes, and genetic changes such as impairment of DNA repair ability, which are caused by DNA damage. It is also reported that inflammation and oxidative stress induced by inhaled chemicals cause DNA damage.^15^ It is necessary, therefore, to observe DNA damage induced by pulmonary inflammation in order to evaluate the potential carcinogenicity of toner with external additives.

We previously performed intratracheal instillation and inhalation exposure using toner with external additives.^16^, ^17^ A 6‐month observation of an intratracheal instillation of toner revealed that persistent inflammation was induced at 2 mg doses.^16^ Although 22.5 months of inhalation exposure did not induce significant lung tumor, 8‐OHdG, a DNA damage marker, increased in a high‐exposure group.^17^ This increase in 8‐OHdG was considered to possibly be a preliminary stage in tumor development from the toner. In the present study, we conducted a 24‐month observation of rats following intratracheal instillation of the same toner which used in our previous studies in order to evaluate the possibility of tumorigenesis caused by toner with external additives.

## 2 MATERIALS AND METHODS

### 2.1 Toner

A test toner was provided by Fuji Xerox Co., Ltd., Tokyo, Japan, as an experimental toner sample to be used exclusively in this study, and is not yet commercially available. The toner was synthesized by dispersed toner components in the liquid phase and covered mechanically and electrostatically with TiO_2_ nanoparticles and amorphous silica nanoparticles as external additives.

### 2.2 Animals

Female RccHan®️: WIST (Wistar Hannover) rats (8 weeks old) were purchased from Japan SLC, Inc. All procedures and animal handling were done according to the guidelines described in the Japanese Guide for the Care and Use of Laboratory Animals as approved by the Animal Care and Use Committee, University of Occupational and Environmental Health, Japan.

### 2.3 Intratracheal instillation of toner

The test toner was suspended with 0.4 mL distilled water including 0.1% Tween 80.

About 1 mg (3.3 mg/kg) or 2 mg (6.7 mg/kg) of toner was intratracheally instilled once to Wistar female rats (40 in each group). A negative control group received distilled water including 0.1% Tween 80.

### 2.4 Protocol

Animals that survived were dissected at 24 months after the instillation. Five rats in each group provided bronchoalveolar lavage fluid (BALF), which was collected using a physiological saline that was injected through a cannula inserted through the respiratory tract into the right lung while the left lung was clamped. Seven to 10 mL (different volumes of lavage fluid were based on the ages of the animals) of physiological saline was infused in the right lung at two times. Between 11 and 14 mL of BALF in total was collected. The left lung was inflated and fixed by 4% paraformaldehyde at 25 cm H_2_O pressure. The right lungs of the remaining rats were obtained for histopathology. The liver, kidney, spleen, and brain were measured in the rats from which BALF samples were collected, while the lung weight was measured in the rats from which BALF samples were not collected. Rats that had deceased during the experimental period were also dissected at that time and their lungs were obtained for histopathology.

### 2.5 Analysis of inflammatory cells in BALF with cytospin

Four hundred grams of the obtained BALF was centrifuged at 4°C for 15 minutes, and the supernatant was transferred to a new tube and frozen for measuring the cytokines. The pellets were washed by suspension with polymorphonuclear leukocyte (PMN) Buffer (137.9 mmol/L NaCl, 2.7 mmol/L KCl, 8.2 mmol/L Na_2_HPO_4_, 1.5 mmol/L KH_2_PO_4_, and 5.6 mmol/L C_6_H_12_O_6_) and 400 g was centrifuged at 4°C for 15 min. After the supernatant was removed, the pellets were resuspended with 1 mL of PMN Buffer. The number of cells in the BALF was counted by Celltac (Nihon Kohden Corp.), and the cells were splashed on a slide glass using cytospin. After the cells were fixed and stained with Diff‐Quik (Sysmex Corp.), the number of total cells, neutrophils, lymphocytes, and alveolar macrophages were counted by microscopic observation.

### 2.6 Measurement of myeloperoxidase (MPO) and lactate dehydrogenase (LDH) in BALF

The concentrations of rat MPO proteins in the BALF samples in all of the examinations were measured by ELISA kits, HK105 (Hycult Biotech, The Netherlands). The activity of LDH in the BALF was measured by a Cytotoxicity Detection Kit^PLUS^ (LDH) (Roche Diagnostics GmbH). All measurements were performed according to the manufacturers’ instructions.

### 2.7 Histopathology

The obtained lung tissue, which was inflated and fixed with 10% formaldehyde or 4% paraformaldehyde under a pressure of 25 cm water, was embedded in paraffin, and 5‐mm‐thick sections were cut from the lobe, then stained with hematoxylin and eosin. DNA damage was evaluated by 8‐hydroxyguanosine (8‐OHdG) and phosphorylated H2AX (γ‐H2AX) immunostaining using the lung tissue samples. Positive controls of hematoxylin and eosin staining or the immunostainings were used lung tissues obtained from the previous inhalation exposure to the same toner of high concentration (16 mg/m^3^) for 22.5 months.^17^

### 2.8 Statistical analysis

Analysis of variance (ANOVA) and Dunnett's test were applied where appropriate to determine individual differences using a computer statistical package (SPSS, SPSS Inc). Kaplan‐Meier survival curves were used to analyze overall survival, with the log‐rank test used to compare different groups. Tumor incidence was analyzed using Fisher's exact test. The level of significance was set at *P* ≤ .05.

## 3 RESULTS

### 3.1 Characterization of toner

**Figure 1:**
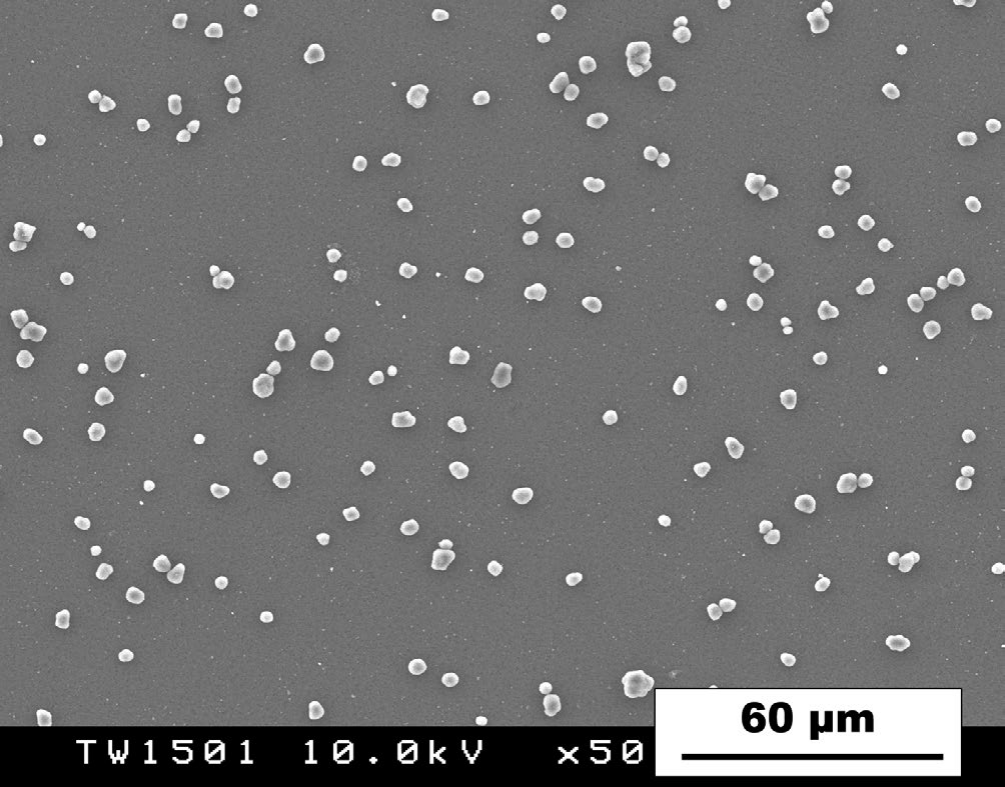
Scanning electron microscopy image of the toners dispersed in the testing suspension

The fundamental characteristics of the bulk toners are summarized in Table 1. The main components of the toner were polyester resin, carbon black, and wax. The total amount of particles smaller than 100 nm in the external additive was 4‐5%, and the main nanoparticles were titanium dioxide and amorphous silica. Figure 1 shows the scanning electron microscopy of the toners with the external additive in the testing suspension. Following the intratracheal instillation study, we examined the harmful effect on the lung of the toner with the external additive.

**Table 1:** Physicochemical properties of toner

Test sample	Physicochemical properties	Value
Bulk toner^a^	Color	Black
	*Component* (*wt%*)	
	Polyester Resin	70‐80%
	Carbon black	1‐10%
	Wax	1‐10%
	Copper compound	1‐10%
	Size by scanning electron microscope	4.05 µm
	Total amount of particles with <100 nm in external additive (wt%)	4‐5%
	Total amount of titanium dioxide nanoparticles in external additive (wt%)	1‐2%
	Total amount of amorphous silica nanoparticles in external additive (wt%)	3‐4%
	BET surface area^b^ (m^2^/g)	2‐3

^a^ Toner: Powder used in laser printers and photocopies to form the printed text and images on paper.

^b^ BET surface area: Brunauer–Emmett–Teller surface area.

### 3.2 Survival rates

**Figure 2:**
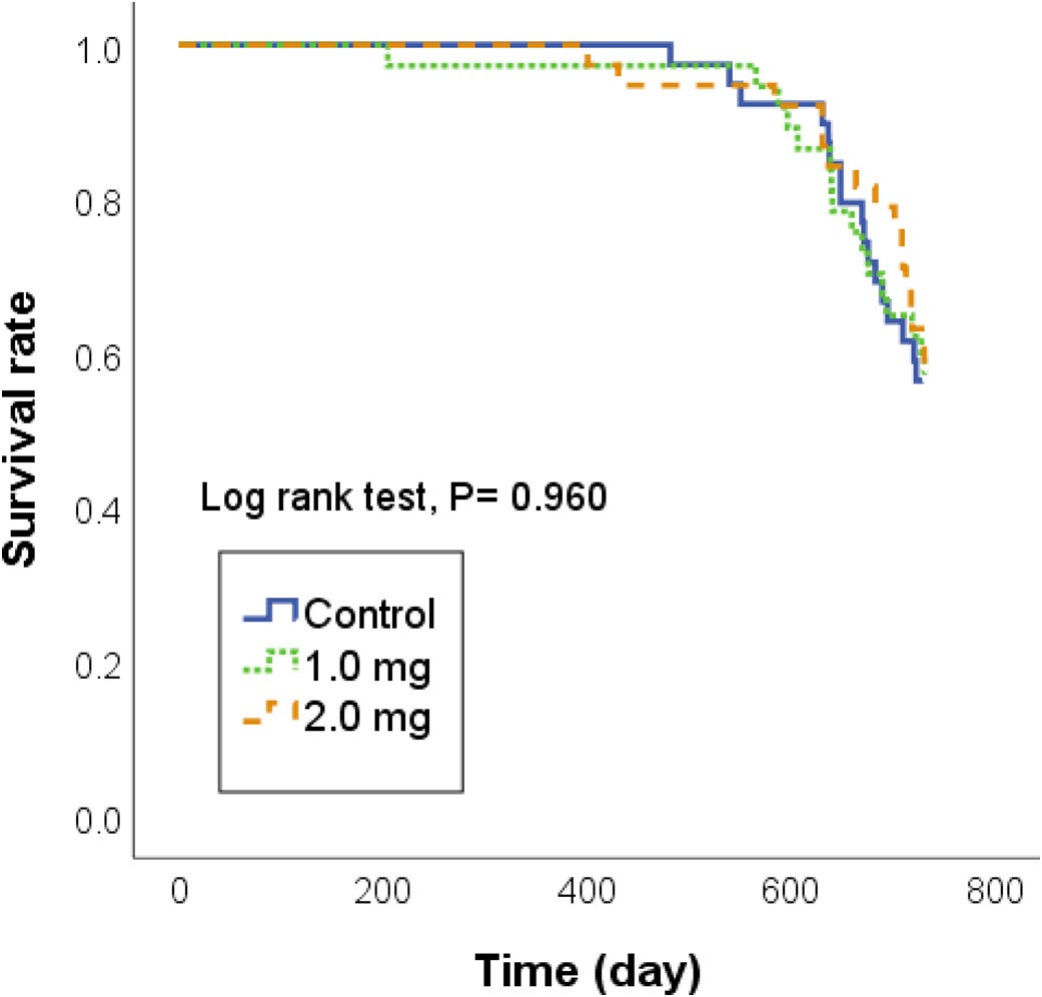
Kaplan‐Meier survival curve analyses of 24 months of observation following intratracheal instillation of toner with external additives. Survival rates were not significantly different in a dose‐dependent manner

Kaplan‐Meier survival curve analysis was performed to evaluate the survival rate after toner exposure in a dose‐dependent manner (Figure 2). Toner exposure was not associated with overall survival (log‐rank test; *P* = .960).

### 3.3 Organ weights

We examined the weight of the body, lung, liver, kidney, spleen, and brain to evaluate the influence of 24 months of toner exposure. There was no significant difference compared with the negative control. Since the weight of the liver, kidney, spleen, and brain also showed no significant change compared to the negative control, we concluded that toner exposure had no influence on those weights (data not shown).

### 3.4 Cell counts in BALF

We measured the inflammatory cells in the BALF to investigate whether the toner induced persistent inflammation (Table 2A). In the 2 mg exposure group, the number of total cells and macrophages showed a statistically significant increase in 24 months of exposure compared to the control. A tendency of increased neutrophil counts was observed in the toner exposure groups, but it was not statistically significant.

**Table 2:** The results of cell counts and inflammatory marker in bronchoalveolar lavage fluid

	Negative control	1.0 mg	2.0 mg
(A) Cell counts (×1000 cells/mL)			
Total cells	114 ± 39.9	174 ± 56.5	197 ± 42.5*
Neutrophils	0.89 ± 1.99	1.67 ± 1.74	1.69 ± 1.23
Lymphoctes	5.09 ± 2.37	7.19 ± 2.47	11.9 ± 6.11
Macrophages	108 ± 38.9	165 ± 56.4	183 ± 35.6*
(B) Inflammatory markers			
MPO (ng/mL)	22.5 ± 28.2	17.1 ± 12.1	17.8 ± 14.5
LDH (U/L)	185 ± 66.4	232 ± 114	200 ± 40.4

Note

Asterisks indicate significant differences compared with negative control (ANOVA, Dunnett T3) (*P < .05, **P < .01).

### 3.5 LDH activity and MPO protein concentration in BALF

We examined the concentration of MPO protein and the activity of LDH in the BALF to evaluate any pulmonary inflammation caused by the toner (Table 2B). There was no increase in MPO or LDH in the exposure groups after 24 months compared to the control group.

### 3.6 Histopathology

**Figure 3:**
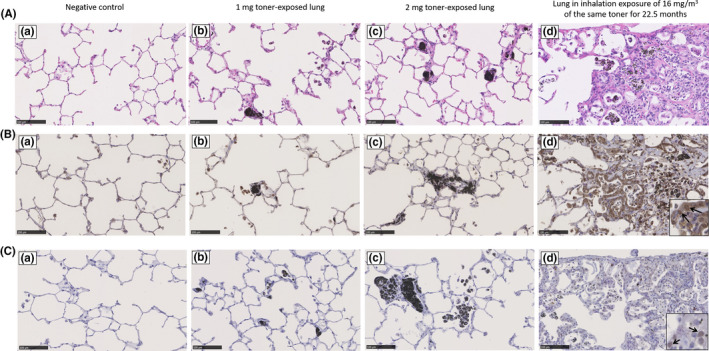
(A) Hematoxylin and eosin staining of lung sections; lung of negative control at 24 months after intratracheal instillation (a), 1 mg toner‐exposed lung at 24 months after intratracheal instillation of toner (b), 2 mg toner‐exposed lung at 24 months after intratracheal instillation of toner (c), and the lung in inhalation exposure of 16 mg/m^3^ of the same toner for 22.5 months as a positive control (d). (B) 8‐OHdG immunostaining of lung sections; lung of negative control at 24 months after instillation (a), 1 mg toner‐exposed lung at 24 months after instillation of toner (b), 2 mg toner‐exposed lung at 24 months after instillation of toner (c), and the lung in inhalation exposure of 16 mg/m^3^ of the same toner for 22.5 months as a positive control (d). (C) γ‐H2AX immunostaining of lung sections; lung of negative control at 24 months after instillation (a), 1 mg toner‐exposed lung at 24 months after instillation of toner (b), 2 mg toner‐exposed lung at 24 months after instillation of toner (c), and the lung in inhalation exposure of 16 mg/m^3^ of the same toner for 22.5 months as a positive control (d)

We investigated the pathological findings by hematoxylin and eosin staining in the lung exposed to toner with external additives (Figure 3A(a‐c)). High‐inhalation toner exposed lung at 22.5 months was showed as a positive control (Figure 3A(d)). Pathological findings in the 1 mg and 2 mg exposure groups at 24 months showed toner particle‐laden alveolar macrophages in the alveoli, but there was no hyperplasia/fibrosis of the bronchoalveolar epithelium. There was a lung tumor in only one sample in the 2 mg exposure group at 24 months. The deceased rats had 4 and 1 lung tumors in the control and 2 mg exposure groups, respectively (Table 3), but these incidences of lung tumor were not statistically significant. The results of 8‐OHdG and γ‐H2AX immunostaining are shown in Figure 3B,C). 8‐OHdG positive was observed in the nucleus of alveolar epithelial cells in the high‐inhalation toner exposure group as a positive control (Figure 3B(d): black arrows). Although there was no clear nuclear staining of the alveolar epithelial cells in the exposure groups, nuclear staining of the alveolar macrophages was observed in the control group and toner intratracheal instillation group. On the other hand, γ‐H2AX positive was observed in the nucleus of some of the alveolar epithelial cells in the positive control group (Figure 3C(d): black arrows), but nuclear staining of the alveolar epithelial cells in the exposure groups was negative.

**Table 3:** The result of lung tumor incidence rates

	Survival	Death	Tumor incidence Survival group (N)	Tumor incidence Death group (N)	Total tumor incidence (N)	*P* value
Control	21	18	0 (0)	22.2 (4)	10.2 (4)	.139
1.0 mg	21	16	0 (0)	0 (0)	0 (0)	
2.0 mg	23	16	4.3 (1)	6.3 (1)	5.1 (2)	

## 4 DISCUSSION

In the present study, rats were intratracheally instilled with toner with external additives and were observed for 24 months. One and two mg of intratracheal instillation were used. In exposure tests of inhalable chemicals, it is necessary to pay attention to overload, when the influence exceeds the toxicity of the chemical itself. There is a report that a clearance delay of toner in rat lung was caused when the amount of toner deposit was more than 1 mg^3^. Another report suggested that 0.4 mg of deposited toner (10 mg/m^3^) did not change the clearance of toner in rat lung, but 3 mg (40 mg/m^3^) did delay the clearance.^18^ From these data, we speculated that exposure to doses in excess of 1–3 mg/rat might induce not only pulmonary toxicity by the chemicals themselves but also toxicity from the excessive dose. In this study, in order to evaluate the toxicity of toner with external additives properly, the intratracheal instillation doses were set 1 and 2 mg as a measure of toxicity from the toner itself and as an estimated borderline of overload, respectively. We used the following formula to estimate what amount in human exposure corresponds to the maximum dose (2 mg) of intratracheal instillation in rats.

$$\begin{array}{cc} \left(\text{amount}\;\text{of}\;\text{toner}\right) & =(\text{exposure}\;\text{concentration}\;\text{of}\;\text{particle})\\ & \;\times (\text{tidal}\;\text{volume})\\ & \;\times (\text{breathing}\;\text{frequency})\\ & \;\times (\text{exposure}\;\text{hours}\;\text{in}\;\text{one}\;\text{day})\\ & \;\times (\text{particle}\;\text{deposition}\;\text{fraction}). \end{array}$$

Assuming that inhaled chemicals would be deposited in rats and humans at the same rate (particle deposition efficiency 0.1, amount of deposited material/1 g of lung weight), we estimated that the exposure time per human would be approximately 3.6 years (calculation in rat and human under the assumption of weight of lung 2 g and 1000 g; tidal volume 2.1 and 625 mL/times; breathing frequency volume 102 and 12 times/min; working time 8 h/d, 5 d/wk), if 2 mg/rat as the lung burden was converted into human exposure at a concentration of 3 mg/m^3^, which the American Conference of Governmental Industrial Hygienists (ACGIH) defined as the threshold limit values–time‐weighted average (TLV‐TWA) of respirable dust.

In the observation of the BALF and in the histopathological findings in this study, there were macrophages with phagocytes of black deposits which could be seen as toner. Although there was an increase in macrophages in the BALF in the 2 mg exposure group, no significant lung inflammation or fibrosis was observed. We previously performed an intratracheal instillation with a toner with external additives and observed focal infiltrations in alveolar macrophages with pigment deposition in the alveoli and persistent inflammation in a 2 mg exposure group from 3 days to 6 months.^16^ In the present study, alveolar macrophages with pigment deposition in the pathological findings and an increase in macrophages in the BALF were observed at 24 months after instillation; therefore, 2 mg might be the dose that exceeds the clearance of macrophages.

We performed an analysis of histopathological changes, as well as a measurement of MPO and LDH as the inflammatory factor in BALF, in order to evaluate chronic inflammation caused by toner with external additives. Although there was some toner deposited in the alveolar spaces, we did not observe any increase in MPO or LDH in the BALF. It is known that MPO in neutrophils and macrophages works as a defense against foreign bodies and that LDH is a marker of cell injury. A.M Knaapen et al reported that an increase in MPO and LDH is associated with lung disorder,^19^ and we have previously shown in a BALF analysis that MPO is related to the pulmonary toxicity of nanomaterials.^20^ That inflammation might have occurred temporarily after the intratracheal instillation of toner, but it is thought that there was not enough inflammation to affect the lung over a long period of time.

We performed 8‐OHdG and γ‐H2AX immunostaining, which are DNA damage markers, to evaluate the potential carcinogenic effect of the toner. The results showed that the nuclei of some of the macrophages were positive for 8‐OHdG in the toner exposure groups, but it was milder than in the positive control group. No γ‐H2AX nuclear staining was observed in the toner instillation groups. It is known that 8‐OHdG is a marker generated from DNA which has been oxidized by reactive oxygen species.^21^ It is also known that γ‐H2AX reflects severe DNA damage,^22^ since it has been shown to be caused by phosphorylation of histone H2AX in chromatin on both sides of a damaged site upon DNA double‐strand break.^23^ Rittinghausen et al performed an intratracheal instillation using crystalline, amorphous silica, and carbon black, and reported that γ‐H2AX had a better correlation with tumor incidence than did 8‐OHdG.^24^ In the present study, the results of 8‐OHdG staining indicated a possibility of mild DNA damage by exposure to toner, but the staining appeared in macrophages; it did not appear in tracheal and alveolar epithelial cells, which are considered to be involved in the development of lung tumor. The γ‐H2AX staining was negative as well; therefore, we speculate that the toner with external additives did not have enough pulmonary toxicity to cause severe DNA damage leading to carcinogenicity. In the exposure to toner with external additives in this study, there was no difference between the exposure and control groups in the induction of lung tumor, and the results of 8‐OHdG and γ‐H2AX immunostaining support this finding.

In this study, the toner with external additives was intratracheally instilled into rats that were dissected at 24 months after exposure, and fibrosis and tumorigenesis were evaluated as chronic effects of toner. Although alveolar macrophages with pigment deposition in the alveoli were observed, no significant lung inflammation/fibrosis or tumor was observed. Immunostaining with 8‐OHdG and γ‐H2AX did not show a remarkable positive reaction, and DNA damage to lung tissue by toner was considered not to be severe. These data suggest that toner with external additives may not have a high potential to cause lung tumor.

## DISCLOSURE

Approval of the research protocol: N/A. Informed consent: N/A. Registry and the registration no. of the study/trial: N/A. Animal studies: All procedures and animal handling were done according to the guidelines described in the Japanese Guide for the Care and Use of Laboratory Animals as approved by the Animal Care and Use Committee, University of Occupational and Environmental Health, Japan.

## CONFLICT OF INTEREST

The funding for this study was provided by Fuji Xerox Co., Ltd. The funding source had no role in the design, practice, or analysis of this study.

## AUTHOR CONTRIBUTIONS

Authors TT, YM, and HI are responsible for the study design and writing of the manuscript. Authors TT, YM, and KY are responsible for data and analysis. Authors HI, YY, TM, CN, and KW performed the experiments. All the authors read and approved the final manuscript.
